# Breviscapine Participates in the Progression of Prostate Cancer by Inhibiting ZFP91 Expression through Upregulation of MicroRNA-129-5p

**DOI:** 10.1155/2021/1511607

**Published:** 2021-12-08

**Authors:** Jie Yang, Wanjun Jin, Xiaokang Zhang, Pengcheng Chang, Duo Zheng

**Affiliations:** ^1^Department of Urology Surgery, The First Hospital of Lanzhou University, Lanzhou, China; ^2^Gansu Institute for Drug Control, Lanzhou, China

## Abstract

**Objective:**

To investigate the effect of breviscapine (BVP) on the development of prostate cancer and its molecular mechanism.

**Materials and Methods:**

After treatment with breviscapine and microRNA-129-5p, MTT (3-(4,5-dimethylthiazol-2-yl)-2, 5-diphenyl tetrazolium bromide) and cell counting kit-8 (CCK-8) tests were performed to examine the proliferation rate of cells, while Transwell was used to analyze cell migration ability; at the same time, quantitative real-time polymerase chain reaction (qRT-PCR) was applied to detect the expression of microRNA-129-5p and ZFP91 in prostate cancer cells. In addition, the binding of microRNA-129-5p and ZFP91 was confirmed by dual-luciferase reporting assay; meanwhile, cell reverse experiment verified that breviscapine can regulate ZFP91 via upregulating microRNA-129-5p.

**Results:**

The results of MTT, CCK-8, and Transwell experiments demonstrated that breviscapine inhibited the proliferation as well as the migration capacities of PC cells; meanwhile, it upregulated the level of microRNA-129-5p in PC cells while downregulated that of ZFP91. Furthermore, dual-luciferase reporter gene assay verified that ZFP91 was a potential target of microRNA-129-5p. Finally, cell reverse experiment confirmed that breviscapine downregulated ZFP91 expression by upregulating microRNA-129-5p, while downregulation of microRNA-129-5p partially reversed the inhibitory effect of breviscapine on cell proliferation ability.

**Conclusions:**

Breviscapine may inhibit the expression of ZFP91 through upregulating microRNA-129-5p and thus participating in the progression of PC.

## 1. Introduction

Natural existing compounds are regarded as the most promising drugs for the prevention and treatment of cancer. They have various model effects and limited toxicity and can regulate cell proliferation and cell cycle arrest [1–3]. Breviscapine (BVP) is isolated from Chinese herbs and has been shown to exert comprehensive biological and pharmacological effects. The main active components of BVP are baicalein, 4, 5, 6-tetrahydroxy flavone-7-glucoside acid [4]. BVP is often applied for long-term treatment of paralysis in Chinese folk medicine [4]. Therefore, BVP plays an effective role in the treatment of cerebrovascular diseases caused by cerebral infarction, chronic arachnoiditis, and its sequelae [5]. In addition, studies have found that BVP can be used to induce apoptosis and inhibit cell proliferation, thus preventing the progress of various tumors [6]. However, there have been rare studies about the effect of BVP in the development of PC.

MicroRNAs (miRNAs) are a group of endogenous non-coding RNAs with about 22 nucleotides in length. miRNA can regulate various biological processes such as cell proliferation, differentiation, and apoptosis [7]. Abnormal miRNA expression has been found in various human tumor tissues including prostate cancer tissues [8–10]. In addition, abnormal expression of some miRNAs has been proved to be closely related to the drug resistance and metastasis of prostate cancer [11–13]. These miRNAs can be used as biomarkers for predicting the progress of prostate cancer. For example, the level of microRNA-141 in serum of patients with PC is remarkably higher than that of the normal control group, which is easy to be detected and has the potential of tumor markers of PC [14]. Besides, microRNA-18a level is markedly increased in patients with prostate cancer, which is closely correlated with the progress of PC [15]. The above studies suggest that miRNAs can serve as biomarkers for the diagnosis of prostate cancer. A previous study demonstrated that BVP exerted anti-tumor and anti-metastasis roles in prostate cancer by inhibiting PAQR4-mediated PI3K/Akt pathway [16]. However, so far, there is no study on the mechanism of BVP combined with miRNAs in regulating the progression of prostate cancer.

Here, we found that breviscapine can lead to the increase of microRNA-129-5p expression in PC cells. MicroRNA-129-5p could inhibit the proliferation of PC cells. Meanwhile, we found that microRNA-129-5p can directly bind to ZFP91 and inhibit its expression. We hypothesized that breviscapine, as a promoter of microRNA-129-5p expression, may provide new directions for the drug treatment of PC.

## 2. Materials and Methods

### 2.1. Cell Culture

Two PC cell lines, LNCap and PC3, were obtained from the American Type Culture Collection (ATCC) (Manassas, VA, USA) and cultured with Roswell Park Memorial Institute 1640 (RPMI 1640) (HyClone, South Logan, UT, USA) supplemented with 10% fetal bovine serum (FBS) (Gibco, Rockville, MD, USA), 100 *μ*g/mL penicillin, and 100 U/mL streptomycin in a 37°C, 5% CO_2_ incubator.

### 2.2. Cell Transfection

LNCap and PC3 cells were plated in 6-well plates. MicroRNA-129-5p mimics (10 pmol) or microRNA-129-5p inhibitor (15 pmol) and miRNA-NC were transfected into cells to achieve microRNA-129-5p overexpression and knockdown using Lipofectamine 2000 (Invitrogen, Carlsbad, CA, USA) according to the manufacturer's standard protocol.

### 2.3. MTT (3-(4,5-Dimethylthiazol-2-yl)-2, 5-Diphenyl Tetrazolium Bromide) Assay

Transfected cells were plated in 96-well plates (1.0 × 10^3^ per well) and were then treated with breviscapine (20, 40, and 80 *μ*g/mL) overnight. Then, 0.5 mg/ml MTT (Sigma-Aldrich, St. Louis, MO, USA) was added to each well. After 4 hours, the medium in each well was replaced with 100 *μ*L of formazan solubilization solution. The plate was gently mixed for 10 minutes so that the formazan crystals could be fully dissolved. Lastly, the optical density (OD) value of each well was detected in the microplate reader (BioTek Instruments, Winooski, VT, USA) at 450 nm absorption wavelength.

### 2.4. Cell Counting Kit-8 (CCK-8) Assay

Cells were digested, and cell density was adjusted to 3000 cells in 200 *μ*L of medium per well in a 96-well plate. After 24 hours of culture, the corresponding siRNA or inhibitor/mimics or NC was transfected into cells. After the treatment (24, 48, and 72 h, respectively), 10 *μ*L of CCK-8 reagent (Dojindo, Kumamoto, Japan) was added. After 20 minutes, the OD value of each well was detected by a microplate reader at 450 nm.

### 2.5. Transwell Assay

Transfected prostate cancer cells (density of 1 × 10^5^ cells/mL) were resuspended in serum-free medium and added to Transwell's upper chamber plate. The lower chamber of Transwell was added with RPMI 1640 containing 10% FBS. The staining and quantity of the migrated cells were determined by randomly selecting the average of five visual field counts under an optical microscope.

### 2.6. Quantitative Real-Time Polymerase Chain Reaction (qRT-PCR)

Total RNA was extracted with TRIzol reagent (Invitrogen, Carlsbad, CA, USA). Complementary deoxyribose nucleic acid (cDNA) was synthesized using a reverse transcription kit (Thermo Fisher Scientific, Waltham, MA, USA). MicroRNA-129-5p expression was examined by real-time quantitative PCR detection, with U6 used as an internal control. The 2^−ΔΔCt^ method was applied to reflect the difference in expression between the target group and the control group. The primer sequences are as follows: microRNA-129-5p (f), 5′-CGGCGGTTTTTTGCGGTCTGGGCT-3′, microRNA-129-5p (r), 5′-AGCCCAGACCGCAAAAAACCGCCG-3′; U6 (f) 5′-CTCGCTTCGGCAGAACA-3′, U6 (r), 5′-ACGCTTCACGAATTTGCGT-3′; GAPDH (f) 5′-GAAGAGAGAGACCCTCACGCTG-3′, glyceraldehyde-3-phosphate dehydrogenase (GAPDH) (r) 5′-ACTGTGAGGAGGGGAGATTCAGT-3′; ZFP91 (f) 5′-TGAGACCTACAAACCCCACTT-3′, ZFP91 (r) 5′-CCTTTTGGGTAAACGTGGACTTT-3′.

### 2.7. Dual-Luciferase Reporter Gene Assay

The binding relationship of microRNA-129-5p to ZFP91 was evaluated by a dual-luciferase reporter experiment. To achieve this, the sequence of ZFP91 (including the binding site of microRNA-129-5p (ZFP91-WT) and the ZFP91 mutant (ZFP91-MUT) containing mismatched microRNA-129-5p binding sequences) was cloned into pmirGLO vector (Promega, Madison, WI, USA). Then, microRNA-129-5p mimics were co-transfected into LNCap and PC3 cells with ZFP91-MUT or ZFP91-WT containing Lipofectamine 2000. Then, the fluorescence intensity was measured. The relative expression of the reporter gene was calculated according to the reporter gene fluorescence intensity/internal reference fluorescence intensity.

### 2.8. Statistical Analysis

All statistical analyses were performed using Statistical Product and Service Solutions (SPSS) software version 22.0 (IBM, Armonk, NY, USA). Significant differences between data were calculated using a paired two-tailed Student's *t*-test or chi-square test. All measurement data are expressed as mean ± SD (standard deviation). *P* < 0.05 was considered statistically different.

## 3. Results

### 3.1. Breviscapine Inhibits Proliferation and Migration of Prostate Cancer Cells

**Figure 1:**
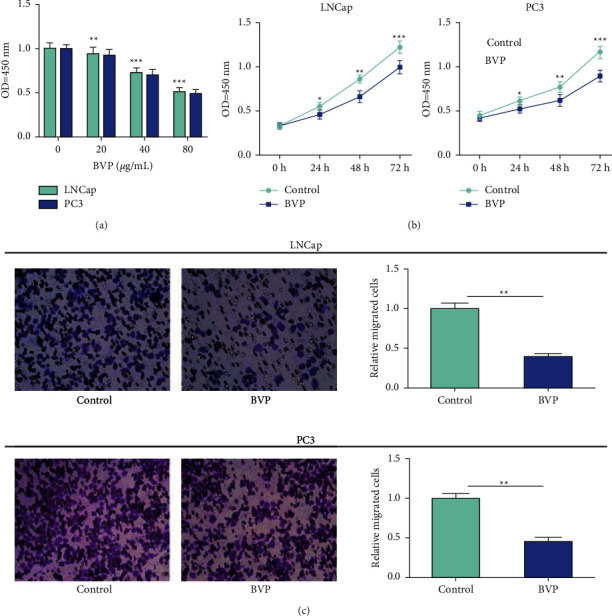
Breviscapine inhibits proliferation and migration of prostate cancer cells. (a) After treatment with 20, 40, and 80 *μ*g/ml breviscapine for 24 h, the cell proliferation ability of LNCap and PC3 cells was significantly inhibited in a concentration-dependent manner. (b) The proliferation ability of cells was significantly inhibited after treatment of LNCap and PC3 cells with 40 *μ*g/mL breviscapine. (c) Treatment with 40 *μ*g/mL breviscapine significantly inhibited the migration ability of LNCap and PC3 cells (^*∗*^*P* < 0.05, ^*∗∗*^*P* < 0.01, and ^*∗∗∗*^*P* < 0.001).

In this experiment, in order to detect whether breviscapine has an effect on PC cell proliferation, cells were administered with different concentrations of breviscapine, and MTT assay was performed 24 hours later. The results of MTT assay showed that breviscapine markedly inhibited the proliferation of PC cell lines *in vitro* with dose dependence (Figure 1(a)). At the same time, we carried out the subsequent CCK-8 and Transwell experiments, which showed that breviscapine markedly inhibited the proliferative and migrant ability of LNCap and PC3 cells (Figures 1(b) and 1(c)). These results demonstrated that breviscapine inhibits the proliferative and migrant ability of PC cells.

### 3.2. Breviscapine Promotes Expression of MicroRNA-129-5p

**Figure 2:**
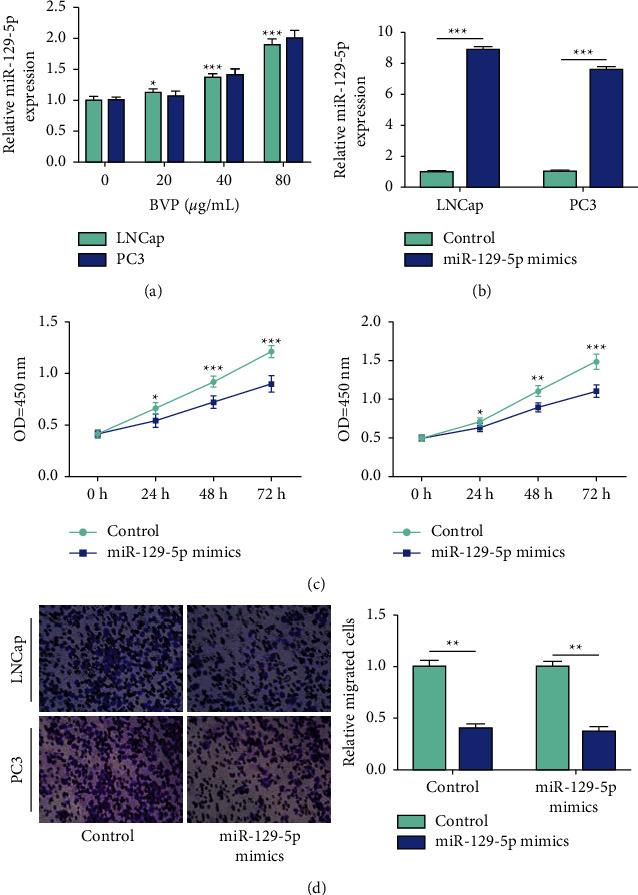
Breviscapine promotes the expression of miR-129-5p. (a) After treatment of LNCap and PC3 cells with 20, 40, and 80 *μ*g/mL breviscapine, the expression of miR-129-5p was significantly increased in a concentration-dependent manner. (b) After transfection of miR-129-5p mimics in LNCap and PC3 cells, the expression of miR-129-5p was significantly increased. (c, d) After upregulating the expression of miR-129-5p in LNCap and PC3 cells, the cell proliferation and migration ability was significantly inhibited (^*∗*^*P* < 0.05, ^*∗∗*^*P* < 0.01, and ^*∗∗∗*^*P* < 0.001).

MicroRNA-129-5p expression was detected by qRT-PCR under different concentrations of breviscapine (0, 20, 40, and 80 *μ*g/mL). We found that the level of miR-129-5p was remarkably elevated by breviscapine in a dose-dependent way (Figure 2(a)). At the same time, in order to detect whether microRNA-129-5p was involved in the progression of prostate cancer cells, we overexpressed miR-129-5p and its negative control into LNCap and PC3 cells, and its expression was significantly upregulated (Figure 2(b)). The CCK-8 results suggested that the proliferation of LNCap and PC3 cells was significantly reduced after microRNA-129-5p overexpression (Figure 2(c)). At the same time, Transwell experiments showed that the migrant ability of LNCap and PC3 cells was remarkably decreased after microRNA-129-5p overexpression (Figure 2(d)). This indicated that microRNA-129-5p may play a tumor suppressing role in PC.

### 3.3. ZFP91 Is a Potential Target of MicroRNA-129-5p

**Figure 3:**
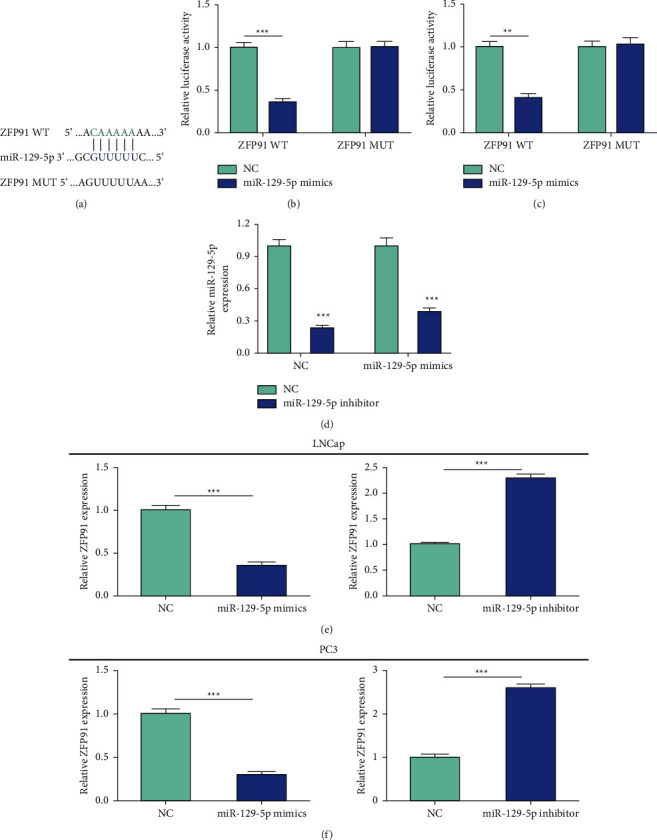
ZFP91 is a potential target gene for miR-129-5p. (a) Bioinformatics analysis revealed that miR-129-5p has a potential binding site with ZFP91. (b, c) The results of the dual-luciferase reporter gene assay confirmed the binding relationship between the two. (d) After transfection of miR-129-5p inhibitor in LNCap and PC3 cells, miR-129-5p expression was significantly reduced. (e, f) The mRNA level of ZFP91 was significantly decreased after upregulating the expression of miR-129-5p in LNCap and PC3 cells; the mRNA level of ZFP91 was significantly increased after downregulating the expression of miR-129-5p (^*∗*^*P* < 0.05, ^*∗∗*^*P* < 0.01, and ^*∗∗∗*^*P* < 0.001).

By database prediction, we identified ZFP91 as a possible target for microRNA-129-5p (Figure 3(a)). To further clarify that this sequence was the target sequence of microRNA-129-5p on ZFP91 mRNA, we constructed the ZFP91 mRNA 3′UTR into the luciferase reporter system and named it ZFP91-WT, and the mutation binding site was also built into the luciferase reporting system and was named ZFP91-MUT. MicroRNA-129-5p mimics were co-transfected with an empty control vector (empty vector) and ZFP91-WT or ZFP91-MUT. The results showed that in LNCap and PC3 cells, the fluorescence expression of the ZFP91- WT + miR-159-5p group was markedly lower than that of the ZFP91-WT + NC group, while the fluorescence intensity of the ZFP91-MUT + miR-159-5p group was correlated with ZFP91-MUT (Figures 3(a)–3(c)). This result indicated that microRNA-129-5p can bind to ZFP91. After transfection of the microRNA-129-5p inhibitor in LNCap and PC3 cells, microRNA-129-5p expression was markedly reduced (Figure 3(d)). To investigate the regulation of ZFP91 expression by microRNA-129-5p at the cellular level, we transfected microRNA-129-5p inhibitor/mimics and negative control (NC) into LNCap and PC3 cells, respectively, and detected the expression of ZFP91by qRT-PCR. The results suggested that ZFP91 expression was markedly decreased after transfection with microRNA-129-5p mimics, while ZFP91 expression was markedly increased after microRNA-129-5p inhibition (Figures 3(e) and 3(f)). The above results indicated that ZFP91 was a potential target gene of microRNA-129-5p.

### 3.4. Breviscapine Inhibits ZFP91 by Upregulating MicroRNA-129-5p

**Figure 4:**
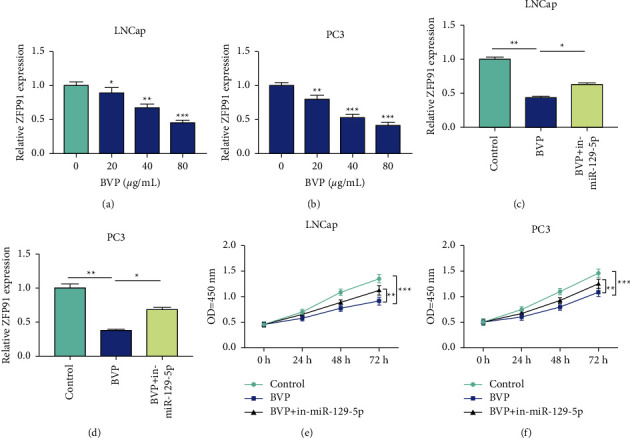
Breviscapine inhibits the expression of ZFP91 by upregulating miR-129-5p. (a, b) After treatment of LNCap and PC3 cells with 20, 40, and 80 *μ*g/ml breviscapine, the expression of ZFP91 was significantly decreased in a concentration-dependent manner. (c, d) Simultaneous inhibition of miR-129-5p expression in LNCap and PC3 cells treated with breviscapine at a concentration of 40 *μ*g/mL partially reversed the inhibitory effect of breviscapine on ZFP91 expression. (e, f) Simultaneously downregulating the expression of miR-129-5p partially reversed the inhibitory effect of breviscapine on the proliferation of LNCap and PC3 cells (^*∗*^*P* < 0.05, ^*∗∗*^*P* < 0.01, and ^*∗∗∗*^*P* < 0.001).

To investigate whether breviscapine could also affect the expression of ZFP91, we examined the expression of ZFP91 after treatment with breviscapine. We found that the expression of ZFP91 was markedly reduced in a concentration-dependent manner, which indicated that breviscapine can inhibit the expression of ZFP91 (Figures 4(a) and 4(b)). Similarly, downregulation of microRNA-129-5p expression in PC cells treated with 80 *μ*g of breviscapine partially reversed the inhibitory effect of breviscapine on ZFP91 expression (Figures 4(c) and 4(d)). Further CCK-8 experiments showed that simultaneous downregulation of microRNA-129-5p partially reversed the inhibition of breviscapine on cell proliferation (Figures 4(e) and 4(f)). In combination with the above experiments, breviscapine may exert anti-cancer effects by upregulating the expression of microRNA-129-5p and inhibiting ZFP91.

## 4. Discussion

Prostate cancer (PC) remains the most common cancer in men worldwide and the second leading cause of cancer-related deaths in the US. In the past decade, the mortality rate of PC has markedly decreased due to the improvement of prostate cancer screening [17]. Serum prostate-specific antigen (PSA) has been used to monitor the progression or recurrence of prostate cancer after treatment. However, patients with prostatitis, benign prostatic hyperplasia, and urinary tract infection can also exhibit elevated serum PSA [18]. Therefore, we should search for biomarkers with higher cancer specificity or a combination of several biomarkers. Understanding the molecular mechanism of prostate cancer and finding more effective treatment strategies for PC is of great importance.

Breviscapine, as a new derivative of traditional Chinese medicine, was proved to inhibit the proliferative and migrant ability of PC cells in this study. In order to further explore the mechanism of its action, we further studied and found that its mechanism may be related to microRNA-129-5p and ZFP91. MicroRNA-129-5p has been recognized as a tumor suppressor for glioblastoma, breast cancer, colorectal cancer, and so on [19–21]. MicroRNA-129-5p is one of the most significantly downregulated miRNAs in PC cells and participates in cell metabolism and proliferation regulation by inhibiting key proteins in the carnitine cycle [22]. In this study, our experiment showed that microRNA-129-5p, as an anti-cancer gene for prostate cancer, was upregulated under the action of breviscapine and inhibited the proliferation and migration of tumor cells.

Several oncogenes, including PAK5 and RET, have been identified as target genes for microRNA-129-5p [23, 24]. However, the regulation between microRNA-129-5p and ZFP91 has not been studied. It has been reported that ZFP91 plays an oncogene role in various tumors, including gastric cancer, pancreatic cancer, and colon cancer [25–27]. More importantly, ZFP91 has also been shown to be an oncogene for prostate cancer [28]. Here, we proved that ZFP91 was negatively regulated by the tumor suppressor microRNA-129-5p in PC cells through dual-luciferase reporter gene experiment, PCR, and functional experiments. Combined with previous studies, we determined that breviscapine played a role by promoting the expression of microRNA-129-5p and negatively regulating the prostate cancer oncogene ZFP91. This study still has some limitations. For example, we did not verify the above conclusions in animal models. In the future, we will perfect some animal experiments to verify some of the regulatory effects and mechanisms of BVP on the occurrence and development of prostate cancer in vivo.

## 5. Conclusions

In conclusion, our study showed that breviscapine can inhibit the process of prostate cancer. Breviscapine inhibited the proliferative and migrant ability of PC cells by upregulating the level of tumor suppressor microRNA-129-5p, thereby inhibiting the expression of its downstream target gene ZFP91. The discovery of breviscapine/microRNA-129-5p/ZFP91 axis not only provides a new target for the treatment of PC but also provides a new perspective for a deeper understanding of the pathogenesis of PC.

## Data Availability

The datasets used and analyzed during the current study are available from the corresponding author upon reasonable request.
